# Near‐Infrared Electroluminescent Conjugated Copolymer: Triphenyalmine‐Functionalized Benzothiadiazole‐Thiophene System for Circularly Polarized OLEDs

**DOI:** 10.1002/marc.202401110

**Published:** 2025-03-05

**Authors:** Benedetta Maria Squeo, Alessia Arrigoni, Francesco Zinna, Lorenzo Di Bari, Chiara Botta, Mariacecilia Pasini, Umberto Giovanella

**Affiliations:** ^1^ Consiglio Nazionale delle Ricerche Istituto di Scienze e Tecnologie Chimiche “Giulio Natta” (CNR‐SCITEC) via A. Corti 12 Milano Italy; ^2^ Dipartimento di Chimica e Chimica Industriale Università di Pisa via Moruzzi 13 Pisa Italy

**Keywords:** Circularly polarized emission, Low bandgap copolymers, Near infrared electroluminescence, OLEDs, R5011

## Abstract

We present a conjugated copolymer designed as a near‐infrared (NIR) emitter for organic light‐emitting diodes (NIR‐OLEDs). The low bandgap donor‐acceptor copolymer, comprising a terthiophene derivative (3T) and benzothiadiazole (BT), is end‐capped with bulky triphenyalmine (TPA) groups (3TBT‐TPA). Compared with the parent homologue capped with simple phenyl rings, 3TBT‐TPA shows enhanced solubility in organic solvents with improved photoluminescence quantum yield thanks to a reduced close packing of chains. 3TBT‐TPA, used both as neat emitting layer and blended with fluorene‐benzothiadiazole (F8BT) host copolymer in a NIR‐OLEDs, shows external quantum efficiency up to 0.58%. Additionally, chiral induction is achieved in the F8BT:3TBT‐TPA blend film using a commercial chiral dopant, yielding circularly polarized (CP), photoluminescence (PL) and electroluminescence (EL) in the range 500–800 nm with a maximum dissymmetry factor in the range of 10^−2^. This is the first example of CP‐NIR EL from a conjugated polymer. This work highlights the potential of TPA‐capped strategy for low bandgap copolymers for efficient NIR‐OLEDs and opens new perspectives in the use of conjugated polymers in NIR photonic applications.

## Graphical abstract

A low bandgap donor‐acceptor emitting copolymer used in NIR‐OLEDs achieves an external quantum efficiency of 0.58%. Through a co‐assembling strategy with a chiral inducer, the first example of circularly polarized near‐infrared electroluminescence (CP‐NIR‐OLED) from a conjugated polymer is presented, promising potential applications in NIR photonics.

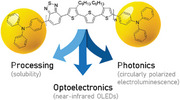

## 1 Introduction

NIR emitters are gathering considerable interest for integration into a variety of applications, ranging from photodynamic therapy to security and defence.^[^ ^1^, ^2^, ^3^ ^]^ Incorporating NIR emitters into OLEDs^[^ ^4^, ^5^, ^6^, ^7^ ^]^ allows these devices to remain small, thin, and flexible, thus reducing their invasiveness and weight, or enabling integration into compact consumer electronics.

One significant challenge is posed by the “energy‐gap law” for radiation less transitions, which suggests that the rate of the non‐radiative transition between two electronic states increases exponentially with a decrease in the energy difference between the states.^[^ ^8^ ^]^ Additionally, NIR emitting dyes frequently encounter issues such as aggregation‐caused quenching due to their extensive π‐conjugated structures.^[^ ^9^, ^10^ ^]^ Large π‐conjugated luminophores often exhibit low solubility and high molecular weight, complicating purification, solution processing, and sublimation.

A careful design of the donor (D) and acceptor (A) units is needed for charge‐transfer D–A emitters, such as thermally activated delayed fluorescence systems, as well as fluorescent copolymers to shift the PL to deep red and NIR. This design must ensure a sufficiently small highest occupied molecular orbital (HOMO)‐lowest unoccupied molecular orbital (LUMO) gap to achieve NIR emission, which requires the introduction of strong D and A groups. However, this strong charge transfer character may result in low oscillator strength or significant solvation‐induced quenching, adversely affecting luminescence performance.^[^ ^4^ ^]^ To achieve nearly pure NIR EL emission (with up to 95% of the overall emission), an effective design strategy has involved the use of copolymers based on benzothiadiazole.^[^ ^11^, ^12^ ^]^

A significant challenge for NIR emitters that would revolutionize NIR‐OLED technology^[^ ^2^, ^4^, ^11^, ^13^, ^14^ ^]^ is the achievement of a circular polarization of the emitted photons.

Identifying suitable organic NIR emitters for CP emitting devices is crucial. Some compounds have shown NIR or deep‐red CPPL including small organic compounds, aggregated systems, and organometallic complexes.^[^ ^15^, ^16^ ^]^ Notably, only a Pt(II) complex and recently a co‐assembly of organic conjugated molecule and a chiral inductor have generated CPEL in NIR‐OLEDs, to date.^[^ ^17^ ^]^ The application of conjugated polymers is still an expectation.

In this work, we present a conjugated copolymer as a NIR electroluminescent emitter. Composed of a terthiophene derivative (3T) as donor and benzothiadiazole (BT) as acceptor, and end‐capped with triphenylamine moieties (TPA) (3TBT‐TPA), it shows good solubility and PL quantum yield (PLQY). When assessed as emitting layer into a device, 3TBT‐TPA emits with EL peaks in the range 730–794 nm. Further, thanks to a co‐assembling strategy, chirality induction in the F8BT:3TBT‐TPA blend is achieved through the incorporation of a chiral inducer and results in CPEL in the range 500–800 nm.

## 2 Results and Discussion

**Figure 1:**
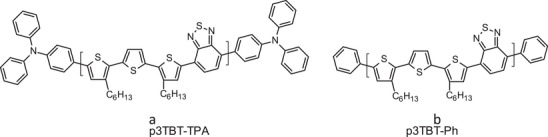
Chemical structures of (a) 3TBT‐TPA and (b) 3TBT‐Ph copolymers.

Polymers 3TBT‐TPA and phenyl‐capped 3TBT (hereafter 3TBT‐Ph, **Figure** **1**b), have been synthetized by Suzuki coupling, using 1:1 monomer feed ratios (Scheme S1). A solution of the commercially available 5,5′“‐dibromo‐3,3′”‐dihexyl‐2,2′:5′,2′'‐terthiophene and 2,1,3‐Benzothiadiazole‐4,7‐bis(boronic acid pinacol ester) were combined in a mixture of degassed toluene and 2 M degassed aqueous solution of K_2_CO_3_ (1.5:1 organic solvent: aqueous solution by volume) in the presence of tetrakis(triphenylphosphine)palladium. The mixture was heated under stirring at 100 °C for 24 h. Polymer 3TBT‐Ph was capped 4‐Bromobenzene and 4‐phenylboronic acid, while polymer 3TBT‐TPA was capped with 4‐bromotriphenylamine and 4‐(diphenylamino)phenylboronic acid according to a consolidated end‐capping procedure.^[^ ^18^, ^19^ ^]^ Purification of the polymers was achieved by Soxhlet extraction with methanol, hexane and toluene. Toluene, that will be used also for film preparation (see below), is preferred to chloroform, which can lead to a residual acidity detrimental for the stability of TPA‐containing polymers. The toluene fractions were then concentrated under reduced pressure, precipitated in methanol, filtered and dried in vacuum, and characterized by ^1^H NMR spectroscopy (Figures S1,S2). The molecular weights (Mw) determined by gel permeation chromatography (GPC) with polystyrene as standard were 1900 g/mol (with PDI 1.6) for 3TBT‐Ph and 5700 g/mol (with PDI 1.2) for 3TBT‐TPA. The difference in chain length can be attributed to the variations in the end‐capping, which involves a first bromine derivative capping, followed 12 hours later by a second capping step with a boronic derivative. The consistency of this difference in chain length was confirmed through simultaneous polymerizations of 3TBT‐Ph and 3TBT‐TPA using the same reagents, freshly distilled solvents, and identical reaction conditions. The reactions were repeated twice, further confirming the reproducibility of the observed polymer lengths.

Notably, the nature of the end‐capping agent can significantly influence the final molecular weight of the polymer. For instance, when phenyl bromide is used as the end‐capper, the polymer chain growth may be quenched due to its high reactivity with boronic acid‐terminated polymer chains, compared to the reactivity of the diboronic starting monomer. This results in shorter polymer chains and a reduced Mw for 3TBT‐Ph. In contrast, the use of TPA bromide as the end‐capper leads to a competitive reaction rate of oxidative addition and trans‐metalation with polymer chain growth, allowing for the incorporation of more monomer units before chain termination. The reduced reactivity of TPA bromide can be attributed not only to its strong electron‐donating nature but also to its steric hindrance, which slows its insertion into the catalytic cycle and provides additional time for chain elongation. As a matter of fact, the 3TBT‐TPA polymer demonstrates enhanced solubility in toluene and superior film‐forming properties due to its increased Mw compared to 3TBT‐Ph.

**Figure 2:**
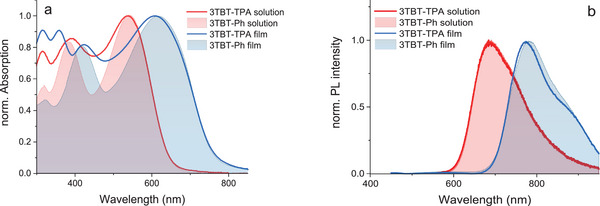
Optical properties. (a) absorption and (b) PL spectra of 3TBT‐TPA (coloured lines) and 3TBT‐Ph (filled areas) solutions (red) and films (blue).

Absorption spectrum of 3TBT‐TPA in toluene solution (**Figure** **2**a and Figure S3a) is characterized by a main band peaked at 543 nm while in solid‐state, the maximum absorption is red‐shifted to 610 nm (**Table** **1**). The main absorption bands are very similar to 3TBT‐Ph copolymer. Similar behavior is observed for PL spectra (Figure 2b, Table S1, and Figure S3b,c). Main PL emission bands of 3TBT‐TPA solution and films are located at 689 and 775 nm, respectively. The slight red‐shift of PL peak (∼8 nm) of 3TBT‐Ph film with respect to 3TBT‐TPA suggests a possible contribution of chain aggregation in the former. In fact, 3TBT‐Ph is poorly soluble in toluene or in other common organic solvents, indicating that TPA bulky groups of 3TBT‐TPA are crucial in enabling solubility and processability of the compound to form high quality film proper for device testing.^[^ ^11^, ^20^, ^21^ ^]^ Notably, although the 3TBT‐TPA features higher Mw, its optical properties, such as the position of the absorption and emission spectra, are similar to those of the shorter 3TBT‐Ph (Figure 2). This suggests that the effective conjugation length in these systems is already close to being maximized, even with a relatively low number of repeating units.

**Table 1:** Summary of optical properties of 3TBT‐Ph, 3TBT‐TPA polymers in toluene solutions and as spin coated films, and films of 3TBT‐TPA blended with F8BT and chiral inducer: absorption (abs) and PL peaks, PLQY.

		abs_MAX_/ nm	PL_MAX_/ nm	PLQY^a^ ^)^
3TBT‐Ph	Solution	547	689	30
	Film	615	783	/
3TBT‐TPA	Solution	543	689	30
	Film	610	775	4
F8BT:3TBT‐TPA	Film	/	754	12
F8BT:3TBT‐TPA:R5011	Film	/	710	14

^a)^ *λ*_exc_ = 525 nm for the solutions and neat polymer films, *λ*_exc_ = 450 nm for the blended film (excitation of the F8BT polymer).

HOMO and LUMO energy levels of 3TBT‐TPA, obtained from cyclic voltammetry analysis, are −3.02 and −4.98 eV respectively (Table S2 and Figure S4). The oxidation peak observed at 0.25 eV (calculated from the onset) and 0.44 eV (calculated from the peak) further confirms the presence of TPA, consistent with findings reported in the literature.^[^ ^22^, ^23^ ^]^

The host‐guest polymer‐polymer approach is explored to investigate possible effects of aggregation on the performance of the copolymer thin film, also in view of the assessment as active layer (AL) in OLED prototypes.

**Figure 3:**
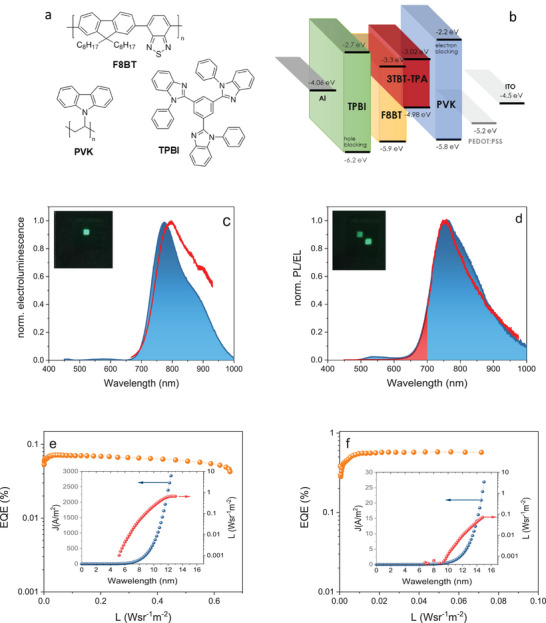
Devices characterization. (a) Molecular structure of the organic materials used in the devices and (b) schematic energy diagram of the NIR‐OLED; PL (blue filled area) and EL (red line) spectra (c,d) (insets, images of devices in operation acquired by means of a NIR visor), (e,f) EQE and JLV characteristics of (c,e) OLED‐N and(d,f) OLED‐D.

The dispersion of 10 wt.% 3TBT‐TPA in F8BT results in a significant increase of PLQY (12%) with respect to neat 3TBT‐TPA film (∼ 4%), accompanied by an obvious blue‐shift of its emission from 775 nm to 754 nm (Table 1 and **Figure** **3**), with a minimal contribution of F8BT emission peaked at 537 nm.

Interestingly, Förster resonance energy transfer (FRET) occurs from F8BT to 3TBT‐TPA. FRET is supported by the spectral overlap between the PL emission of F8BT and the absorption spectrum of 3TBT‐TPA (Figure S5a). Furthermore, the PL excitation (PLE) spectrum of the F8BT:3TBT‐TPA blend film, recorded by monitoring the PL emission at the main 3TBT‐TPA peak (754 nm), closely resembles the absorption spectrum of F8BT (Figure S5b). Such findings are crucial as they suggest potential applications in optoelectronic devices, where energy transfer efficiency can significantly enhance device performance.

Solution‐processed OLEDs were fabricated with neat 3TBT‐TPA (non‐doped) and F8BT: 3TBT‐TPA (doped) AL configurations. To enhance electron/hole balance and exciton confinement within the AL, which are crucial for optimal OLED performance, we employed a multi‐layered architecture with additional functional layers.^[^ ^24^ ^]^ Specifically, a hole‐transporting polymer, polyvinylcarbazole (PVK, Figure 3b), and an electron‐injecting molecule, 2,2′,2′'‐(1,3,5‐Benzinetriyl)‐tris(1‐phenyl‐1‐H‐benzimidazole) (TPBI, Figure 3b), were deposited as thin films using solution and thermal evaporation methods.^[^ ^25^ ^]^ The device structure was as follows: Indium‐Tin Oxide (ITO)/Poly(2,3‐dihydrothieno‐1,4‐dioxin)‐poly(styrenesulfonate) (PEDOT:PSS)/PVK/AL/TPBI/LiF/Al (Figure 3a). These configurations are indicated as OLED‐N for non‐doped and OLED‐D for doped devices, respectively. In OLED‐D the F8BT:3TBT‐TPA mass ratio is fixed to 10:1 since it exhibits best performance (see Figure S6).

All devices predominantly exhibit EL emission from 3TBT‐TPA (Figure 3c,d). The EL peaks are located at 753 nm to 794 nm for doped and non‐doped devices, respectively. Notably, not EL emission from F8BT was detected in OLED‐D architecture. The emission from OLED‐N is characterized by a truly NIR EL, whereas for OLED‐D, approximately 5% of the emission occurs below 700 nm (red filled area in Figure 3d). OLED‐N switches on at 5 V, with a EQE_MAX_ of 0.072% and radiance (L) of 0.68 Wsr^−1^m^−2^ at 12 V (Figure 3e). In the doped configuration, OLED‐D turns on at about 9 V and exhibits enhanced EQE_MAX_ of 0.58% and L of 0.1 Wsr^−1^m^−2^ at 12 V (Figure 3f).

The performance of the devices are comparable to the results published in the literature for all organic NIR‐OLEDs, but they remarkably stand out when considering that the NIR emitter is a conjugated polymer.^[^ ^2^, ^4^, ^5^ ^]^

**Figure 4:**
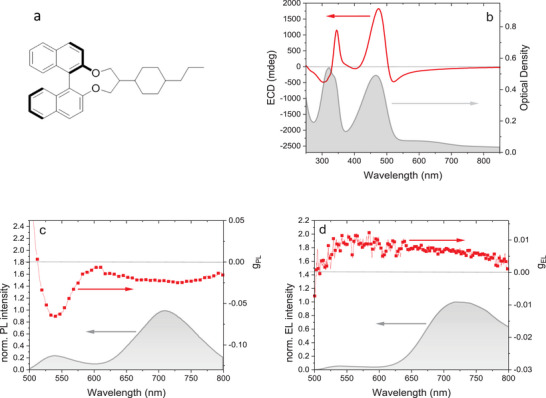
Chiroptical characterization of film and devices embedding R5011 chiral inducer. (a) Chemical structure of the chiral inducer; (b) Absorption and ECD spectra, (c) PL and *g*_PL_ of F8BT:3TBT‐TPA:R5011 film; (d) EL and *g*_EL_ of ITO/PEDOT:PSS/F8BT:3TBT‐TPA:R5011/LiF/Al device.

Recent advancements in chirality induction within conjugated polymers have shown promising results, particularly through chirality transfer between a small chiral inducer and the achiral host polymer, leading to notable CPEL.^[^ ^26^, ^27^, ^28^ ^]^ Expanding on this strategy, we incorporated a commercial chiral inducer, marketed as R5011 (**Figure** **4**a), into the F8BT:3TBT‐TPA co‐assembled system. The goal is to induce chiral characteristics within the F8BT:3TBT‐TPA system, aiming to enhance NIR CPEL.

R5011 was chosen due to its proven chiral induction efficacy to F8BT.^[^ ^28^ ^]^ By co‐assembling it with F8BT:3TBT‐TPA blend, the inducer is expected to interact with polymer chains, creating a helical conformation and producing a chiral environment in the polymer film. The F8BT:3TBT‐TPA:R5011 ternary blend film, with 10:1:2 mass content ratio, was thermally treated at 140 °C for 30 min^[^ ^28^ ^]^ to facilitate chiral self‐organization (Figure S7).

The film exhibited a significant electronic circular dichroism (ECD) signal of approximately 1700 millidegrees at the absorption band of the achiral F8BT (467 nm, Figure 4b), while no significant ECD band is observed for 3TBT‐TPA.

The degree of CP for both PL and EL emission was measured by means of the dissymmetry factor g (g_PL/EL_).^[^ ^29^, ^30^ ^]^ The chiral co‐assembly displayed CPPL signal with g_PL_ of about −0.07 in F8BT emission region between 530–550 nm. Surprisingly, CPPL is observed also for 3TBT‐TPA band with a negative g_PL_ value again in the order of 10^−2^ in the range 550–800 nm (Figure 4c).

Besides its role in chirality induction, the R5011 additive acts as a dispersing agent for F8BT:3TBT‐TPA blend, producing a blue‐shift of PL band associated to 3TBT‐TPA with a minor increase in PLQY (14%) with respect to binary blend. At the same time, a slight increase in the contribution of F8BT emission to the overall PL spectrum (Figure 4c) with respect to F8BT:3TBT‐TPA binary blend is observed.

Corresponding devices are manufactured with the basic architecture ITO/PEDOT:PSS/AL/LiF/Al (OLED‐C).

The integration of the chiral inducer into the system results in a blue shift of the NIR EL spectrum and a slight contribution of F8BT emission relative to the overall EL (Figure 4d). Nevertheless, the devices predominantly exhibit NIR EL, with 75% of the EL spectrum located above 700 nm, achieving an EQE_MAX_ of 0.28% and a L_MAX_ of 3 Wsr^−1^m^−2^ (Figure S8).

Notably, OLED‐C demonstrated CPEL with a maximum *g*_EL_ value of 0.01, that slightly decreases in the NIR region, reaching approximately 5×10^−3^ in correspondence of the 3TBT‐TPA emission band. This represents the first example of CP‐NIR‐OLEDs with CPEL directly generated from a conjugated polymer.

The sign of CPEL is opposite to that of CPPL of corresponding film. This behavior may be rationalized by taking into account the circular self‐extinction within the active layer, due to a significant overlap between ECD bands and the emission profile. Recently, we have developed a model to (semi)‐quantitatively predict the polarization outcoupling (and therefore the g_EL_) of the device, from the g_PL_ profile, cathode reflectance, absorption/ECD spectra of the active layer as well as the position of the recombination zone (see Supporting Information).^[^ ^31^, ^–^, ^33^ ^]^ Applying this model, we can reproduce the sign inversion (Figure S9,10), assuming that hole‐electron recombination occurs close to the cathode. The model is also able to reproduce correctly the order of magnitude of the g_EL_, especially at lower energy.

## 3 Conclusions

In summary, we have successfully developed and characterized a conjugated copolymer, 3TBT‐TPA, designed as a NIR emitter for OLEDs. The introduction of TPA bulky steric groups significantly enhanced solubility and the film‐forming ability with respect to the Ph‐capped copolymer. The NIR‐OLEDs fabricated using 3TBT‐TPA exhibited good EQE of 0.58% with EL peak at 753 nm. Notably, the induction of CPPL and CPEL is demonstrated by following a co‐assembling strategy with a chiral inducer achieving dissymmetry factors of ∼5×10^−3^ for 3TBT‐TPA NIR emission. This represents the first example of CP‐NIR‐OLEDs with directly generated CPEL from a conjugated polymer. The observed inversion in the sign of CPEL compared to CPPL highlights the complex interplay of factors influencing device performance, including the circular self‐extinction within the active layer, the positioning of the radiative exciton recombination zone and the architecture of the device.

These findings open new avenues for the application of conjugated polymers in NIR photonic devices, suggesting a promising future for the development of efficient and versatile CP‐NIR‐OLEDs.

Further studies are needed to fully elucidate the mechanisms behind the observed phenomena and to optimize the performance of these innovative devices.

## 4 Experimental Section

### General Information for synthesis

All reagents were purchased from commercial sources and used without further purification. All solvents have been degassed bubbling nitrogen prior to use. All reactions were carried out in an inert atmosphere.

### Synthesis of 3TBT‐TPA

A mixture of 5,5′“‐dibromo‐3,3′”‐dihexyl‐2,2′:5′,2′'‐terthiophene (250 mg, 0.235 mmol, 1 eq.), 2,1,3‐Benzothiadiazole‐4,7‐bis(boronic acid pinacol ester) (169 mg, 0.235 mmol, 1eq.), tetrakis(triphenylphosphine)palladium (10 mg, 2% mol), and TEBA (benzyltriethylammonium chloride) was added in a pre‐degassed Schlenk flask, followed by three vacuum/nitrogen cycles. Then degassed toluene (9 mL) and potassium carbonate 2 M water solution (2.25 mL) were added. After the mixture was heated under stirring at 100 °C for 24 h, 4‐bromotriphenylamine was added, followed, after 7 h, by 4‐(diphenylamino)phenylboronic acid. After 12 h the mixture was diluted with toluene and filtered on celite pad. The polymer was purified by precipitation in methanol and washed using a Soxhlet apparatus with methanol, hexane and toluene.^[^ ^34^ ^]^ The toluene fraction was concentrated under reduced pressure, precipitated in methanol, filtered, and dried in vacuum to give a purple powder (70 mg, 72% yield).

### Synthesis of 3TBT‐Ph

A mixture of 5,5′“‐dibromo‐3,3′”‐dihexyl‐2,2′:5′,2′'‐terthiophene (148 mg, 0.258 mmol, 1 eq.), 2,1,3‐Benzothiadiazole‐4,7‐bis(boronic acid pinacol ester) (100 mg, 0.258 mmol, 1eq.), tetrakis(triphenylphosphine)palladium (6 mg, 2% mol), and TEBA (benzyltriethylammonium chloride) was added in a pre‐degassed Schlenk flask, followed by three vacuum/nitrogen cycles. Then degassed toluene (6 mL) and potassium carbonate 2 M water solution (1.50 mL) were added. After the mixture was heated under stirring at 100 °C for 24 h, 4‐bromophenyl was added, followed, after 7 h, by 4‐phenylboronic acid. After 12 h the mixture was diluted with toluene and filtered on celite pad. The polymer was purified by precipitation in methanol and washed using a Soxhlet apparatus with methanol, hexane, and toluene.^[^ ^34^ ^]^ The toluene fraction was concentrated under reduced pressure, precipitated in methanol, filtered, and dried in vacuum to give a purple powder (68 mg, 65% yield).

### Chiroptical characterization

Electronic absorption spectra were carried out on a Perkin–Elmer Lambda 900 UV–vis NIR Spectrometer. ECD spectra were recorded with a Jasco J1500 spectropolarimeter. Steady‐state and time‐resolved PL spectra were recorded on a modified Jobin‐Yvon Horiba Fluorolog spectrofluorometer. PLQYs have been obtained with a home‐made integrating sphere. CPPL and CPEL activities are evaluated by means of a fixed linear polarizer (LP) and a rotating quarter wave plate (QWP) placed in front of the CCD detector, as previously described.^[^ ^25^ ^]^ Control measurements were carried out with the home‐built spectrofluoropolarimeter described in ref. [35] using a photo elastic modulator driven at *f* = 50 kHz. The residual fluorescence linear anisotropy component was measured with the same instrument by extracting the 2f signal of the PEM modulation.

### Electrochemical measurements

Electrochemistry was performed at room temperature in acetonitrile under nitrogen in three electrode cells. The counter electrode was platinum; reference electrode was Ag/Ag^+^ (0.1 m AgNO_3_ in acetonitrile, 0.34 V versus SCE, −4.73 V versus vacuum), supporting electrolyte was 0.1 m tetrabutylammonium perchlorate). 3TBT‐TPA film was casted onto electrodes from toluene solution. The working electrode was a glassy‐carbon minidisc electrode (0.2 cm^2^). HOMO and LUMO levels were estimated according, to the Equation *E*_HOMO_ = −(*E*_ox_ + 4.39 + 0.34) and *E*_LUMO_ = −(*E*_Red_ + 4.39 + 0.34).^[^ ^36^ ^]^

### Film and Devices

Solutions of 3TBT‐TPA and F8BT with 10wt.% of 3TBT‐TPA in toluene at a total concentration of 20 mg/mL were spin‐coated on quartz substrates. Solutions of the F8BT:3TBT‐TPA blended with 20wt.% of R5011 in toluene at a total concentration of 20 mg/mL were spin‐coated on glass substrate and annealed at 140 °C for 30 min in inert atmosphere. For the manufacturing of devices indium thin oxide (ITO) was used as anode. After sequential cleaning of 2.5 cm x 2.5 cm sized ITO‐patterned‐coated glasses in acetone and isopropanol in a sonicator, they were treated with nitrogen plasma. PEDOT:PSS Clevios VPI 4083 was spin‐coated to form a ∼30 nm thick film. PVK (∼35 nm) film was deposited on top of PEDOT:PSS from a 10 mg/mL chlorobenzene solution and annealed at 150 °C for 15 min. Solution of the F8BT:3TBT‐TPA:R5011 was spin‐coated on top of the glass/ITO/PEDOT:PSS/PVK substrate. The TPBI electron transport/hole blocking layer (30 nm) was deposited using an Organic Molecular Beam Deposition system. Finally, 1.5 nm of LiF and 110 nm of Al were thermo‐sublimated inside the high vacuum evaporator to achieve a mirror cathode. Photons emitted in a forward direction through the glass substrate were collected by a calibrated photodiode. Current density‐light‐voltage curves were recorded by Keithley 2602 apparatus. The radiance of the devices was estimated using the same setup and Lambertian emission was assumed.

## Conflict of Interest

The authors declare no conflicts of interest.

## Supporting information



Supporting Information

## Data Availability

The data that support the findings of this study are available in the supplementary material of this article.
