# Supplementary material for: Near‐Infrared Electroluminescent Conjugated Copolymer: Triphenyalmine‐Functionalized Benzothiadiazole‐Thiophene System for Circularly Polarized OLEDs
Source: Macromol Rapid Commun. 2025 Mar 5;46(12):2401110. doi: 10.1002/marc.202401110 (PMC12183154; doi:10.1002/marc.202401110)
Supplement: Supplementary file 1 — Supporting Information [file MARC-46-2401110-s001.pdf]

Macromolecular Rapid Communications

Supporting Information

for *Macromol. Rapid Commun.*, DOI 10.1002/marc.202401110

Near-Infrared Electroluminescent Conjugated Copolymer: Triphenyalmine-Functionalized Benzothiadiazole-Thiophene System for Circularly Polarized OLEDs

Benedetta Maria Squeo, Alessia Arrigoni, Francesco Zinna, Lorenzo Di Bari, Chiara Botta, Mariacecilia Pasini and Umberto Giovanella**

Supporting Information

Near-Infrared Electroluminescent Conjugated Copolymer: Triphenyalmine-Functionalized Benzothiadiazole-Thiophene System for Circularly Polarized OLEDs

Benedetta M. Squeo, Alessia Arrigoni, Francesco Zinna, Lorenzo Di Bari, Chiara Botta, Mariacecilia Pasini, Umberto Giovanella**

B.M. Squeo, A. Arrigoni, C. Botta, M. Pasini, U. Giovanella
Consiglio Nazionale delle Ricerche, Istituto di Scienze e Tecnologie Chimiche "Giulio Natta" (CNR-SCITEC), via A. Corti 12, Milano, Italy
F. Zinna, L. Di Bari
Dipartimento di Chimica e Chimica Industriale, Università di Pisa, via Moruzzi 13, Pisa, Italy

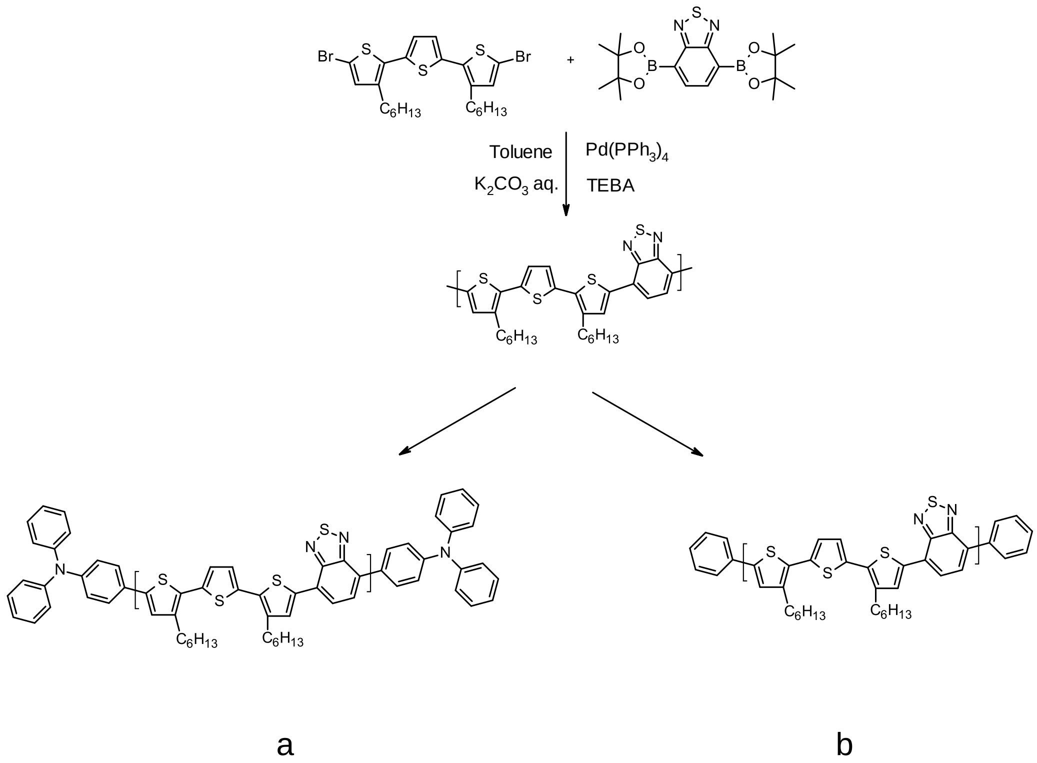

Scheme S1. Synthesis of 3TBT-TPA (a) and 3TBT-Ph end-capped (b) copolymers.

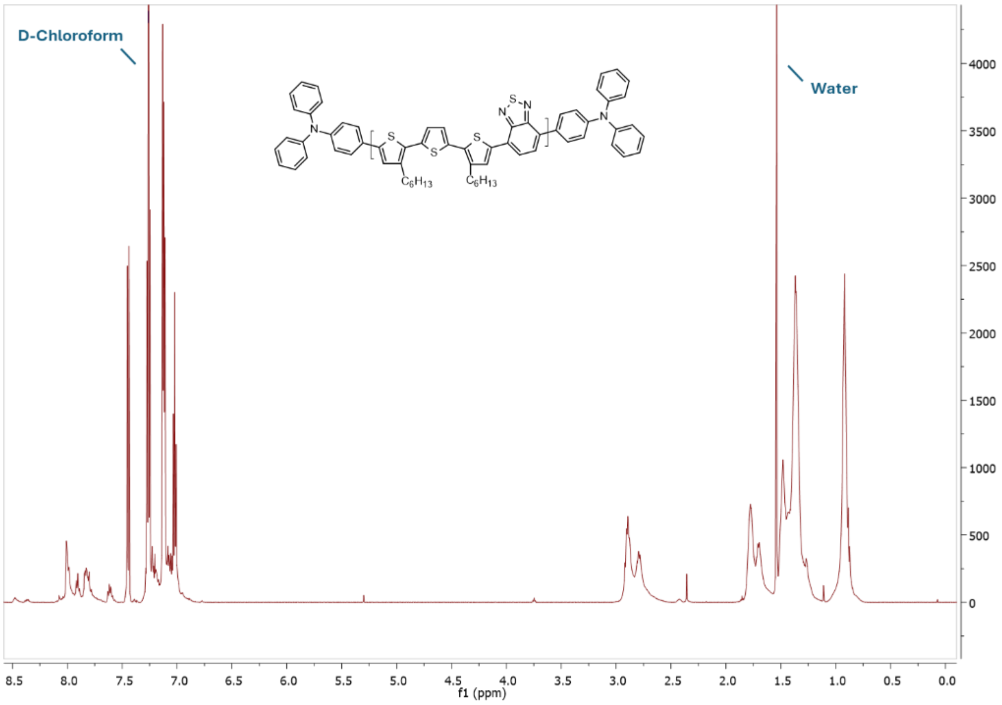

Figure S1. ^{1}H NMR spectrum of 3TBT-TPA end-capped copolymers.

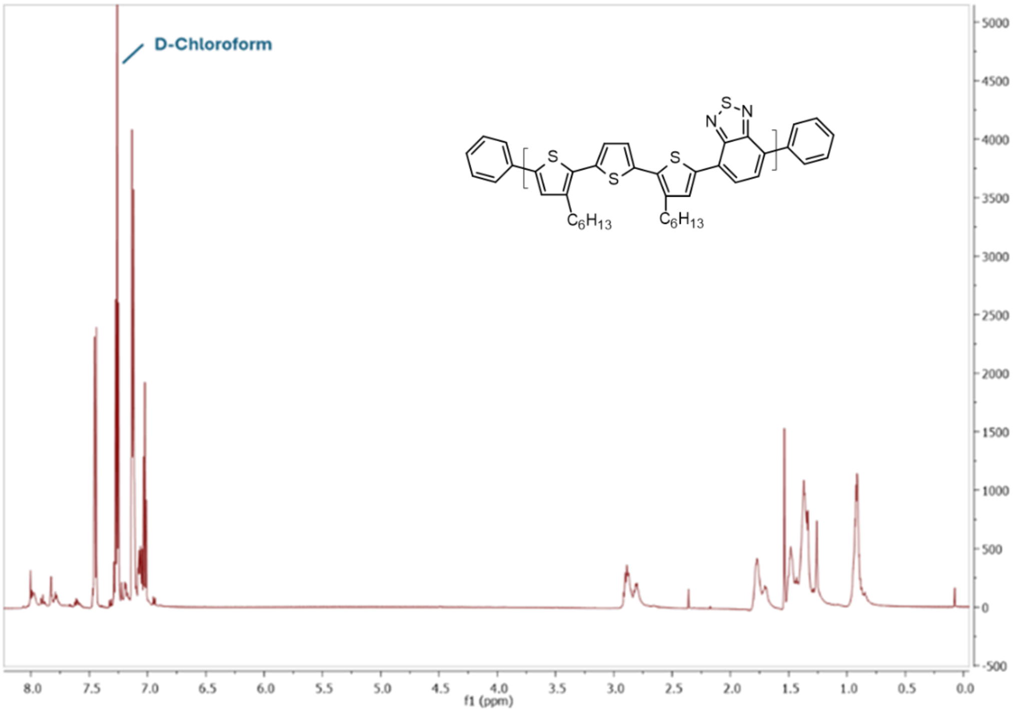

Figure S2. ^{1}H *NMR spectrum of* 3TBT-Ph end-capped copolymers.

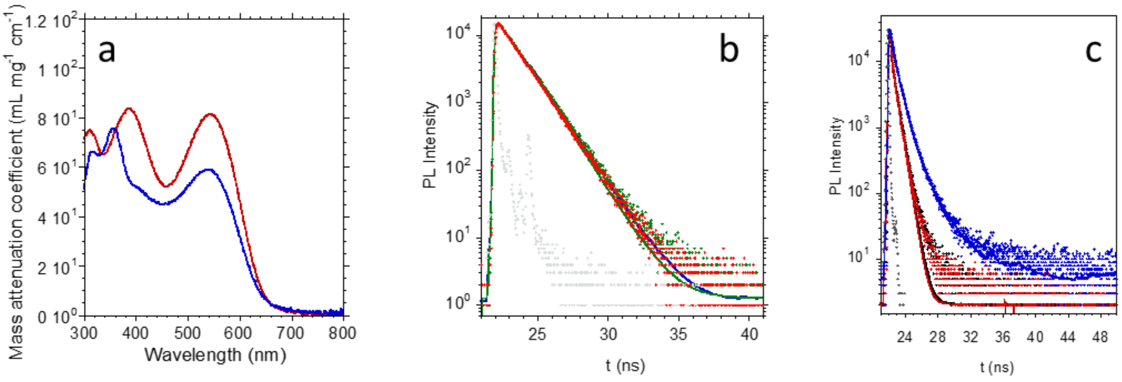

Figure S3. (a) Mass attenuation coefficient [1] versus wavelength of 0.0166 mg/ml toluene solution of 3TBT-Ph (red line) and 3TBT-TPA (blue line) (optical path 0.2 cm), and (b) PL decays of 3TBT-TPA and 3TBT-Ph in Toluene solutions, excitation 408 nm, emission 690 nm, with mono-exponential fits; 3TBT-Ph, green points, fit, blue line: τ =1.355 ns, CHISQ = 1.58737; 3TBT-TPA: red points, fit, green line τ =1.308 ns, CHISQ = 1.676182.; (c) PL decays of spin-coated films of 3TBT-TPA (red points) and 3TBT-Ph (black points), emission 760 nm, and 3TBT-TPA:F8BT (blue points, 740 nm), excitation 408 nm,with best fits with the following parameters: 3TBT-TPA, 2-exponential fit (0.82) 0.07779882 ns; (0.18) 0.5475402 ns, CHISQ = 33.10464; 3TBT-Ph, 2-exponential fit (0.81) 0.08117925 ns, (0.19) 0.5723642 ns, CHISQ = 36.39246; 3TBT-TPA:F8BT, 3-exponential fit (0.64) 0.1325047 ns, (0.34) 0.7187068 ns, (0.02) 2.104553 ns, CHISQ = 8.315743.

Table S1. PL lifetimes τ (from mono-exponential fit) and average lifetimes τ_{av}, (from bi- or tri-exponential fits).

		lifetime (ns)
3TBT-Ph	solution[a)]	1.31
	film[b)]	0.36
3TBT-TPA	solution[a)]	1.35
	film[b)]	0.38
F8BT:3TBT-TPA	film[c)]	0.72

a) τ measured at 690 nm (mono-exponential fit); b) τ_{av} measured at 760 nm (2-exp fit); c) τ_{av} measured at 740 nm (3-exp fit). Average lifetimes are obtained as $\tau_{av} = \sum_i \frac{A_i \tau_i^2}{A_i \tau_i}$

Table S2. Oxidation and reduction potentials of 3TBT-TPA calculated at onset and peak.

E_{ox}^{Onset} (ev)	E_{red}^{Onset} (eV)	E_{ox}^{Peak} (eV)	E_{red}^{Peak} (eV)	E_{opt} (eV)	E_{gap} (eV)
0.44	-1.75	0.25	-1.70	1.65	1.96

The optical band gap (E_{opt}) was calculated from the absorption onset of the thin film (1.65 eV), while the electrochemical band gap (E_{gap}) was derived from the HOMO and LUMO energy levels obtained via CV (1.96

eV). The E_{gap}- E_{opt} difference (~300 meV) is consistent with the exciton binding energy typically observed in organic semiconductors, and supports the reliability of both the optical and electrochemical measurements [2].

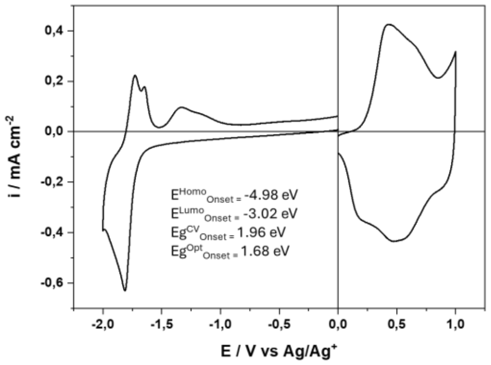

Figure S4. Cyclic Voltammetry plot of 3TBT-TPA.

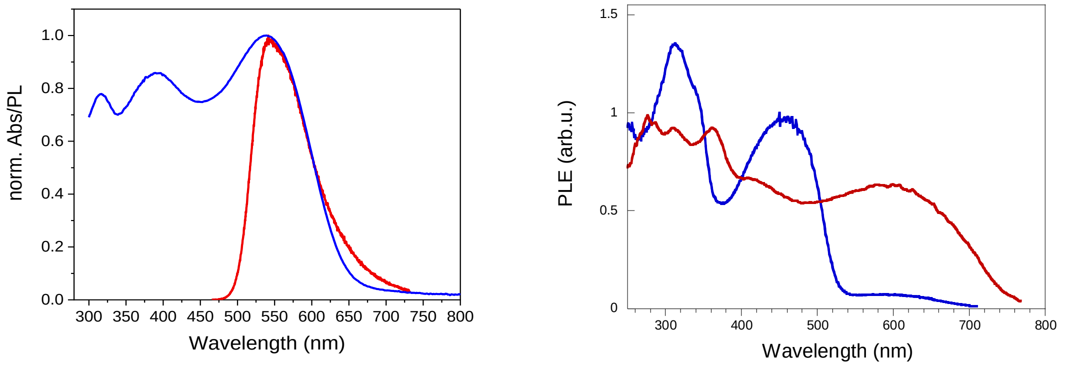

Figure S5. (a) Spectral overlap between UV-Vis-NIR absorption spectrum of 3TBT-TPA (blue line) and PL emission of F8BT (red line); (b) excitation spectra (PLE) by monitoring emission at 725 nm for F8BT:3TBT-TPA blend (blue line) and 745 nm for pure 3TBT-TPA film (red line).

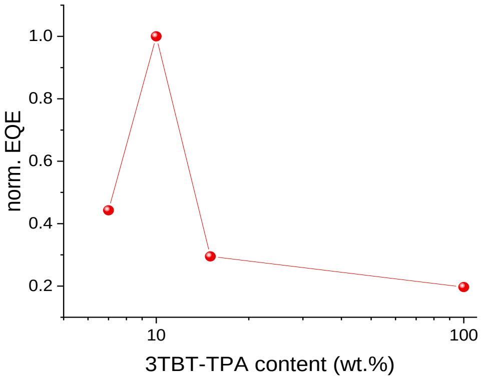

Figure S6. Normalized EQE of OLED-D with different 3TBT-TPA content.

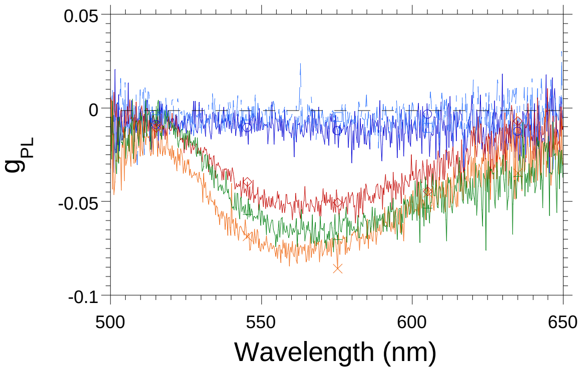

Figure S7. g_{PL} of F8BT films: pristine film (blue), pristine co-assembled film with R5011 chiral inducer (light blue), co-assembled films annealed at 100 °C (red), 140 °C (green) and 150 °C (orange) for 30mins.

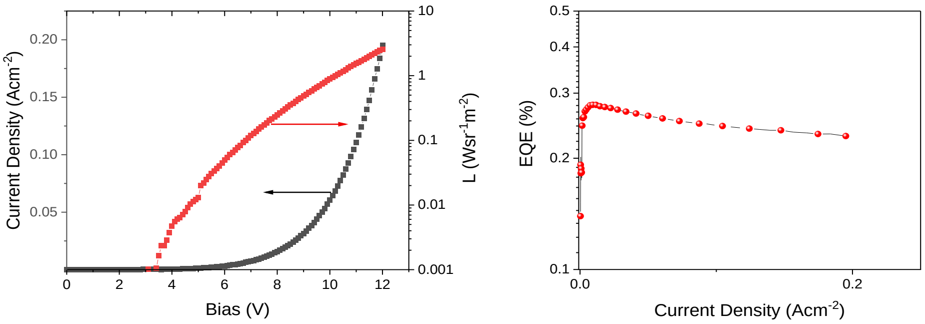

Figure S8. JLV characteristics (a) and EQE plot vs J (b) of OLED-C.

Polarization outcoupling in the CP-OLED. According to our model [3], the electroluminescence dissymmetry factor can be described by:

$$g_{EL} = 2\frac{(1-\gamma 10^{\Delta\alpha\cdot x})-10^{-2\alpha(d-x)}\cdot(10^{\Delta\alpha\cdot x}-\gamma)\cdot R}{(1+\gamma 10^{\Delta\alpha\cdot x})+10^{-2\alpha(d-x)}\cdot(10^{\Delta\alpha\cdot x}+\gamma)\cdot R} \quad \text{eq. S1}$$

With α and Δα being the decadic extinction and differential extinction ($\alpha_L - \alpha_R$) per unit length, R the cathode reflectivity, d the thickness of the active layer, x the position of the recombination zone (RZ) from the anode, and

$$\gamma = \frac{2-g_{PL,corr}}{2+g_{PL,corr}} \quad \text{eq. S2}$$

$g_{PL,corr}$ is the PL dissymmetry factor corrected for circular self-extinction [4], after:

$$g_{PL,corr} \approx g_{PL} + \frac{1}{2}\ln(10)\cdot\Delta A \quad \text{eq. S3}$$

The sign inversion in the CPEL observed for device ITO/PEDOT:PSS/F8BT:3TBT-TPA:R5011/LiF/Al with respect to CPPL can be rationalized by the model in the limit of the RZ fully on the cathode (x = d), with R = 1 (see Figure S8).

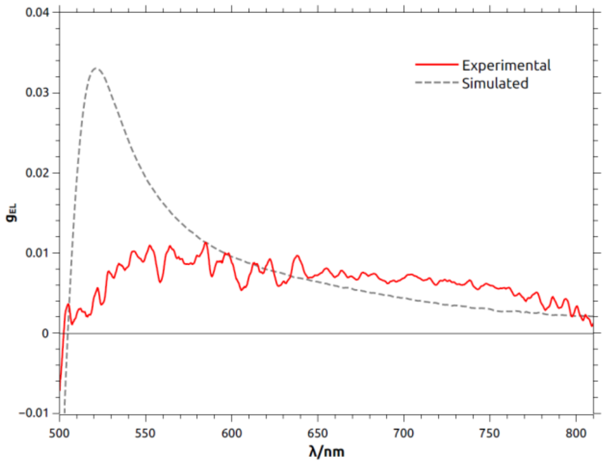

Figure S9. Experimental versus simulated g_{EL}.

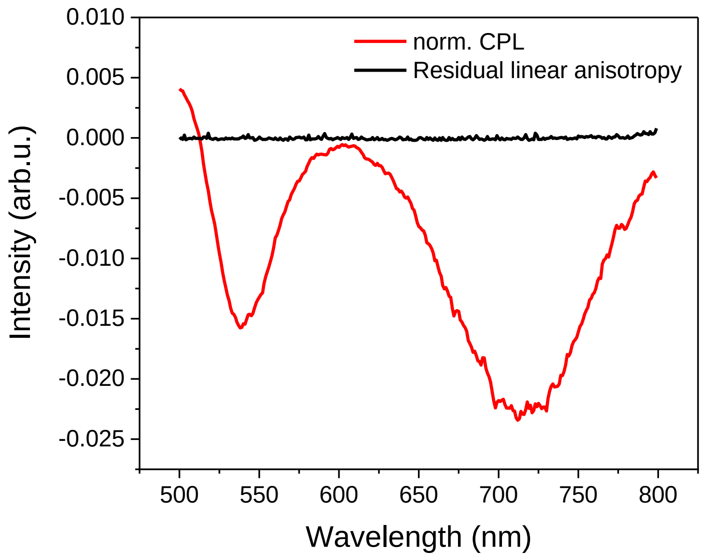

Figure S10. CPPL and residual fluorescence linear anisotropy of F8BT:3TBT-TPA:R5011 film.

REFERENCES

[1] G. Zhang, S. Ma, W. Wang, et al. *Frontiers in Chemistry*, **2019**, 7, Article 359

[2] J.-L. Bredas, *Mater. Horiz.*, **2014**,1, 17-19

[3] F. Zinna, C. Botta, S. Luzzati, L. Di Bari, U. Giovanella, *Adv. Funct. Mater*. **2025**, 2423077.

[4] E. Castiglioni, S. Abbate, F. Lebon, G. Longhi, *Chirality*, **2012**, *25*, 725.
